# Hemodynamically balanced congenitally corrected transposition of the great arteries with a large ventricular septal defect, and subvalvular pulmonic stenosis: a case report

**DOI:** 10.1186/s13256-019-2145-1

**Published:** 2019-07-19

**Authors:** Sang-Yeong Cho, Yeonyee E. Yoon, Wonjae Lee, Si-Hyuck Kang, Young Hwan Song, Cheong Lim, Goo-Yeong Cho, Jeong-Wook Seo

**Affiliations:** 1 Department of Cardiology, Cardiovascular Center, Seoul National University Bundang Hospital, Seongnam, Gyeonggi-do Republic of Korea; 2 Department of Pediatrics, Cardiovascular Center, Seoul National University Bundang Hospital, Seongnam, Republic of Korea; 3 Department of Thoracic and Cardiovascular Surgery, Cardiovascular Center, Seoul National University Bundang Hospital, Seongnam, Republic of Korea; 4 Department of Pathology, Seoul National University College of Medicine, Seoul, Republic of Korea; 5 Department of Cardiology, Gyeongsang National University School of Medicine and Gyeongsang National University Changwon Hospital, Changwon, Republic of Korea

**Keywords:** Congenitally corrected transposition of the great arteries, Ventricular septal defect, Pulmonary hypertension, Straddling mitral valve

## Abstract

**Background:**

Adults with unoperated congenitally corrected transposition of the great arteries are rare but form a distinct group among adults with congenital heart disease. Patients with congenitally corrected transposition of the great arteries often have one or more associated cardiac anomalies that dictate the need for, and timing of, surgical intervention in childhood. However, in a proportion of patients, the hemodynamics does not require surgical attention during childhood, and, in some patients, a correct diagnosis is not established until adulthood. Here we report an adult case of unoperated congenitally corrected transposition of the great arteries with a large ventricular septal defect and probable pulmonary arterial hypertension.

**Case presentation:**

Our patient was a 46-year-old Korean man. Transthoracic echocardiography and cardiac catheterization demonstrated hemodynamically balanced ventricles with a non-regurgitant systemic atrioventricular valve, normal pulmonary arterial pressure, and a reasonable difference between the oxygen saturation values of the aorta and pulmonary trunk, even with the presence of a large ventricular septal defect. Further morphological assessments using cardiac computed tomography and three-dimensional modeling/printing of his heart revealed that the mitral valve was straddling over the posteriorly positioned ventricular septal defect, which could explain the functional and anatomical subvalvular pulmonary stenosis and a small amount of shunt flow through the large ventricular septal defect. We interpreted this combination of cardiac defects as able to sustain his stable cardiac function. Thus, we decided to maintain his unoperated status.

**Conclusion:**

A detailed anatomical understanding based on transthoracic echocardiography, cardiac computed tomography, and three-dimensional printing can justify a decision to not operate in cases of congenitally corrected transposition of the great arteries with hemodynamically balanced pulmonary stenosis and a ventricular septal defect, as observed in the present case.

**Electronic supplementary material:**

The online version of this article (https://doi.org/10.1186/s13256-019-2145-1) contains supplementary material, which is available to authorized users.

## Background

Congenitally corrected transposition of the great arteries (ccTGA) is a complex congenital cardiac anomaly with atrioventricular and ventriculo-arterial discordance [1]. The morphologic right ventricle (RV) functions as a systemic ventricle connected between the left atrium and the aorta, whereas the morphologic left ventricle (LV) functions as the pulmonary ventricle connected between the right atrium and the pulmonary trunk. A morphologic and functional spectrum complicates the clinical presentation, and the nature and extent of associated anomalies dictate the need for, and timing of, surgical intervention. The initial surgical correction is usually applied at a pediatric age, depending on the associated anomalies. In a proportion of patients, however, the hemodynamics does not require surgical attention during childhood, and, in some, the correct diagnosis is not established until adulthood [2]. Adult survivors with operated or unoperated ccTGA are referred to large adult congenital heart disease clinics, and cardiologists often face dilemmas regarding surgical interventions for this infrequent condition.

Here we report an adult case of unoperated ccTGA with a large ventricular septal defect (VSD). Even with the presence of a large VSD, cardiac catheterization demonstrated hemodynamically balanced ventricles with normal pulmonary arterial pressure, and a reasonable difference between the oxygen saturation values of the aorta and pulmonary trunk. Using multimodal imaging, comprising transthoracic echocardiography (TTE), cardiac computed tomography (CCT), and three-dimensional modeling and printing technologies (MEDIP® software, Medical IP Inc.), we evaluated the anatomical substrates of the defect and subsequently decided to maintain medical management until cardiac transplantation was needed in this morphological setting of ccTGA.

## Case presentation

A 46-year-old Korean man, a general office worker, was referred to our institution for the evaluation of congenital heart disease with severe pulmonary arterial hypertension (PAH). He was a social drinker and current tobacco smoker. He had a history of type 2 diabetes controlled with orally administered hypoglycemic agents (metformin, linagliptin, and glimepiride). A cardiac defect was suspected when he was young, but he was asymptomatic and his somatic growth was normal. Detailed assessment of his cardiac lesion was conducted when he was 30-years old. Even after the detection of cardiac defects, he missed routine follow-ups because he was asymptomatic. One month prior to admission, he suddenly developed intermittent chest pain at rest that continued for several minutes. At the primary hospital, he was diagnosed as having a congenital heart disease of VSD, tricuspid regurgitation, and severe PAH; he was transferred to our hospital for further evaluation and management. His general condition was quite good and there was no evidence of neurological or cardiovascular disorder except a pansystolic murmur. His vital signs were as follows: blood pressure of 129/79 mmHg, heart rate of 78 beats/minute, respiratory rate of 18 breaths/minute, and body temperature of 36.6 °C. Electrocardiography demonstrated a sinus rhythm with an incomplete right bundle branch block and bi-atrial abnormality, and a chest X-ray showed mild cardiomegaly. The results of laboratory tests were within normal limits: white blood cells of 6600/μL, hemoglobin of 16.1 g/dL, platelets of 212 k/μL, blood urea nitrogen of 12 mg/dL, serum creatinine of 0.93 mg/dL, aspartate aminotransferase of 28 IU/L, and alanine aminotransferase of 26 IU/L.

**Fig. 1:**
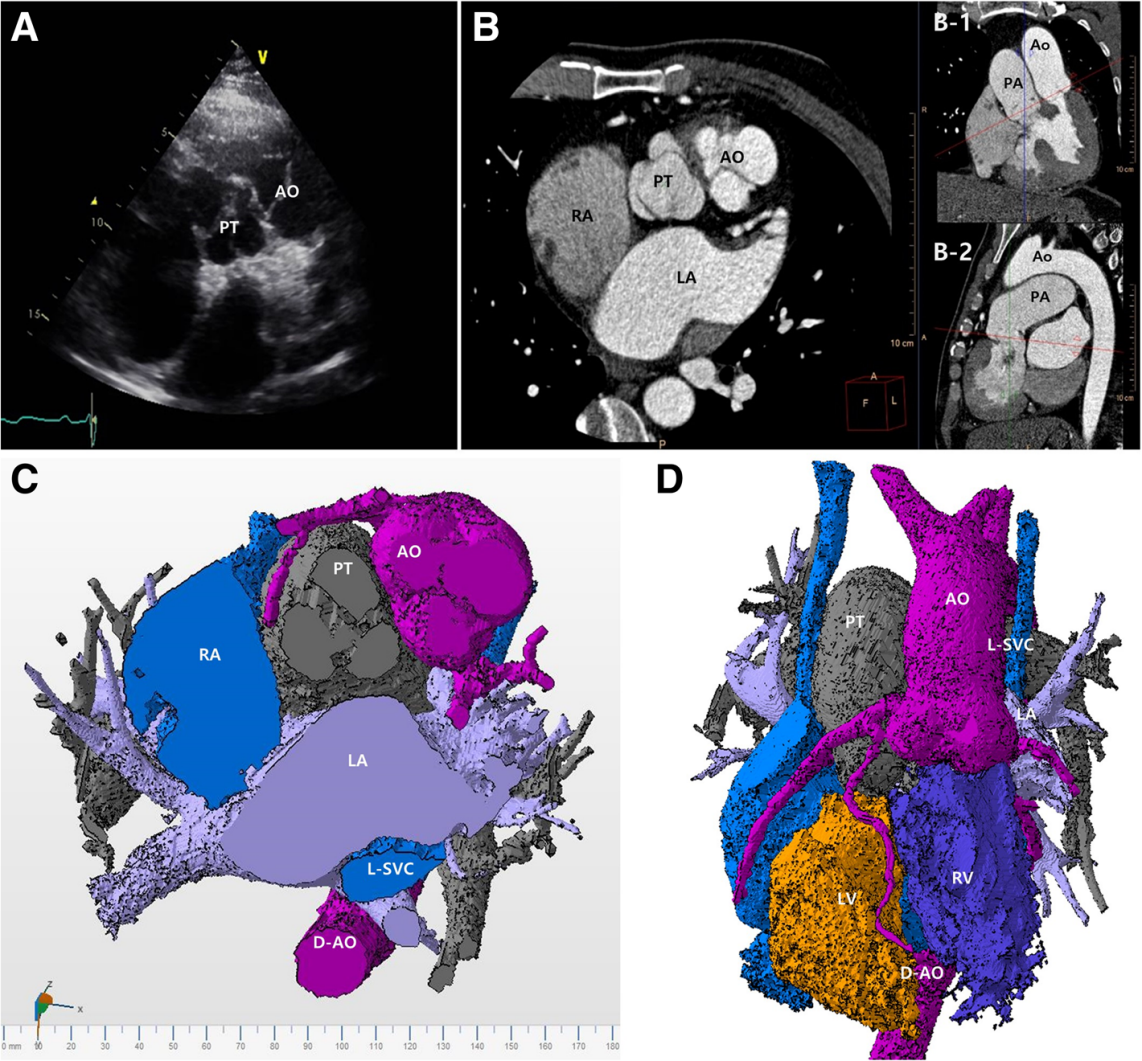
**a** Transthoracic echocardiography shows the pulmonary trunk in the center position of the parasternal short axis view, which was opened to the right-sided ventricle, and the aortic valve in the 1 o’clock position, suggesting the transposition of the aorta and pulmonary trunk. **b** Cardiac computed tomography images at the cutting planes shown in *red lines* in B-1 and B-2 demonstrate the aorta and pulmonary trunk to be in similar positions to those shown by transthoracic echocardiography. **c** The cranial part of the three-dimensional model of the cardiac computed tomography at a similar plane as that in (**a** and **b**). **d** The parallel arrangement of the aorta and pulmonary trunk are shown in a three-dimensional model. *AO* aorta, *D-AO* descending aorta, *LA* left atrium, *L-SVC* left superior vena cava, *LV* left ventricle, *PA* pulmonary artery, *PT* pulmonary trunk, *RA* right atrium, *RV* right ventricle

**Fig. 2:**
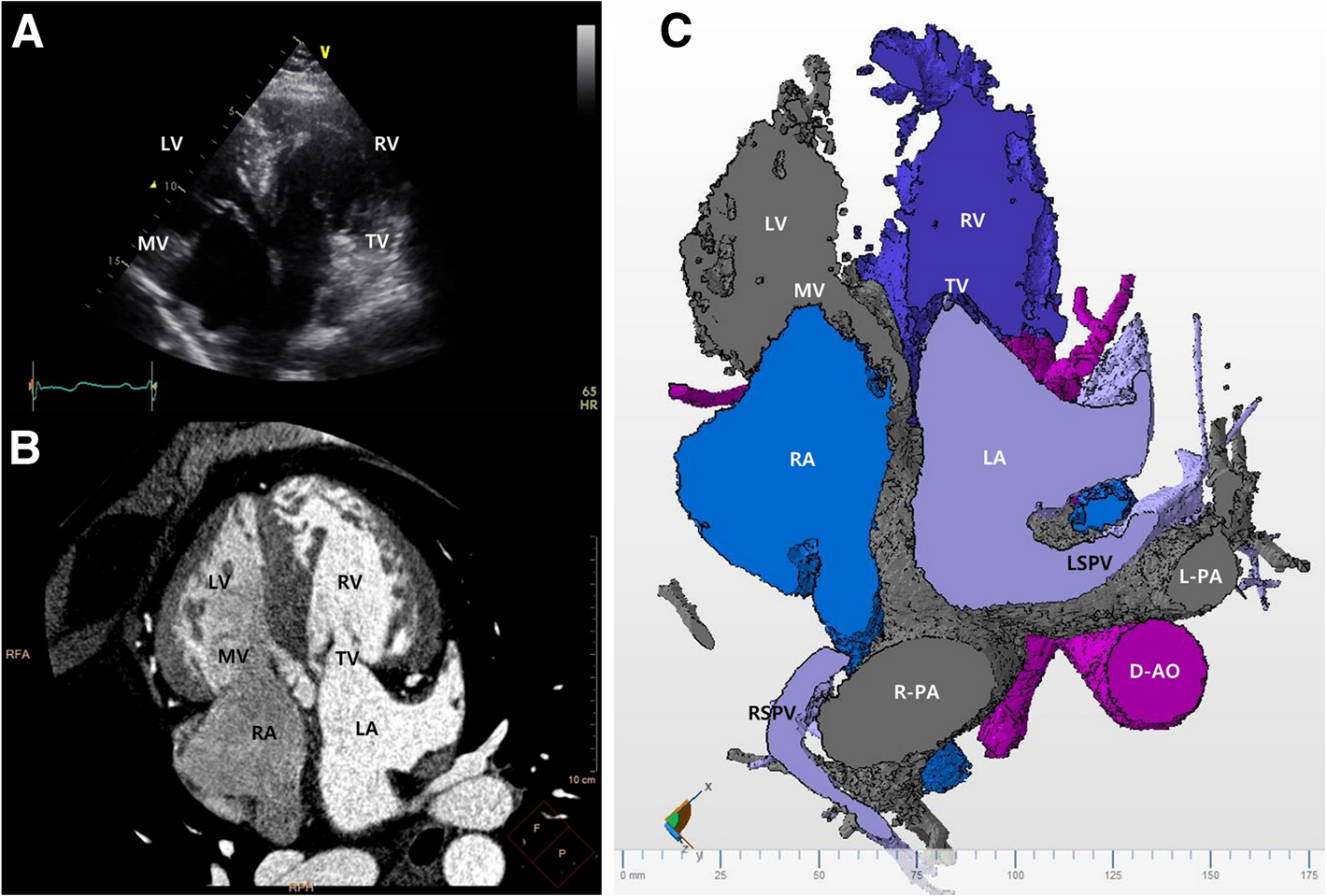
**a** Transthoracic echocardiography in the four-chamber view plane demonstrates that the left-sided systemic ventricle is the morphologically right ventricle. **b** Cardiac computed tomography image in a similar plane to that in (**a**) is shown. The right ventricle has prominently coarse trabeculation and a more apically displaced tricuspid valve than that of the mitral valve. The other smooth-walled right-sided pulmonic ventricle is the morphologically left ventricle. **c** A three-dimensional model of the cardiac computed tomography at a similar plane to that in (**a** and **b**) is shown. *D-AO* descending aorta, *LA* left atrium, *L-PA* left pulmonary artery, *LSPV* left superior pulmonary vein, *LV* left ventricle, *MV* mitral valve, *RA* right atrium, *R-PA* right pulmonary artery, *RSPV* right superior pulmonary vein, *RV* right ventricle, *TV* tricuspid valve

On TTE, the pulmonary valve was at the center position of the parasternal short axis view (Fig. 1a), and was opening well and arising from the right-sided ventricle. The aortic valve was on the left anterior side of the pulmonary valve, suggesting ventriculo-arterial discordance (Fig. 1a). CCT and three-dimensional modeling demonstrated the aorta and pulmonary trunk to be in similar positions to those shown by TTE (Fig. 1b, c), and demonstrated the parallel arrangement of the great arteries (Fig. 1b, d). TTE and CCT showed atrioventricular discordance in addition to ventriculo-arterial discordance (Fig. 2a-c). The left-sided morphologically RV was a systemic ventricle with prominently coarse trabeculation and an apically displaced atrioventricular valve as a tricuspid valve. The other smooth-wall right-sided pulmonic ventricle was morphologically a LV. Three-dimensional modeling of the chamber revealed similar information (Fig. 2c). The ejection fraction of the left-sided systemic ventricle (RV) was 58%.

**Fig. 3:**
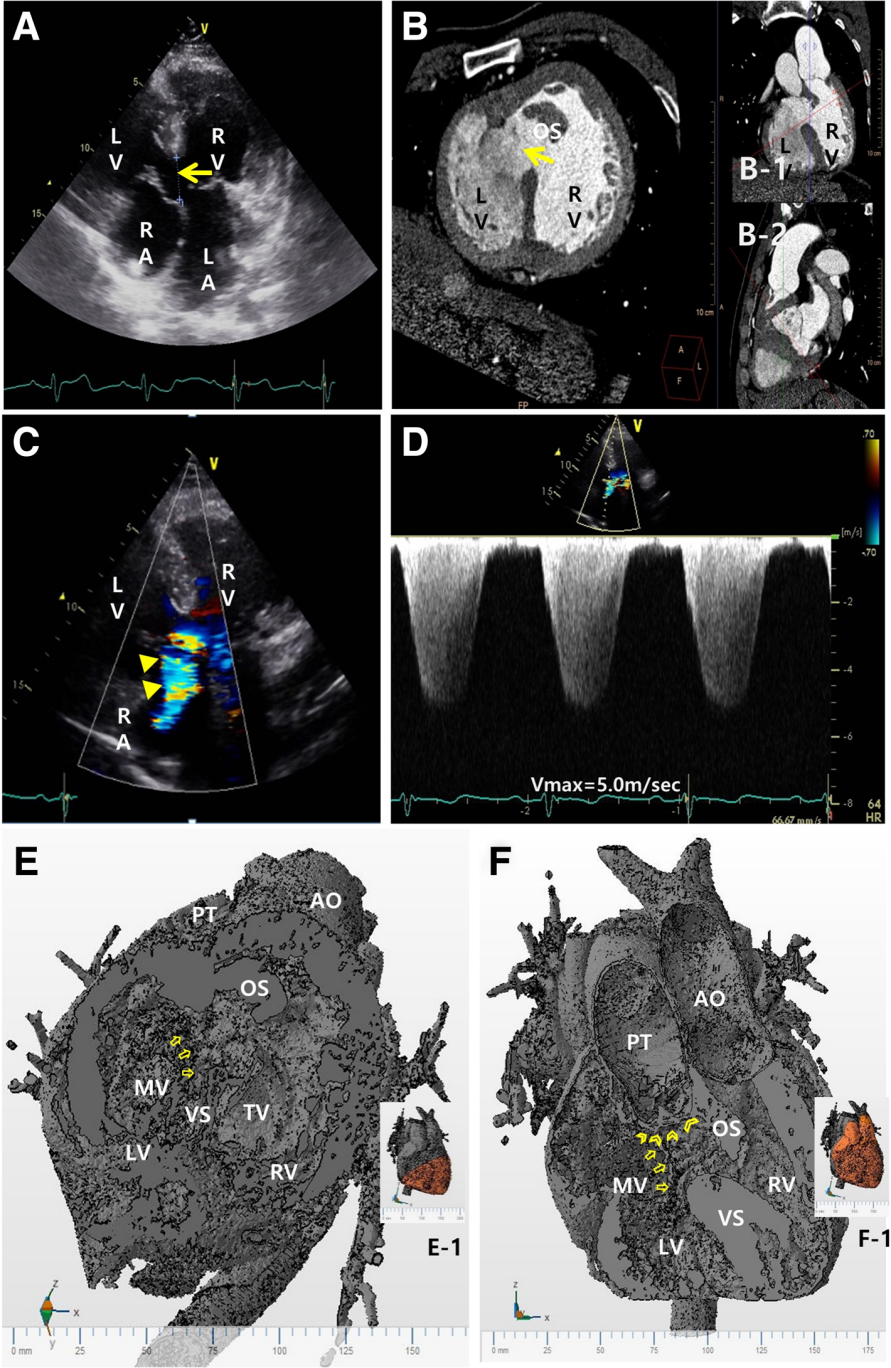
**a** Apical four-chamber view on transthoracic echocardiography shows a large ventricular septal defect (*arrow*). **b** Cardiac computed tomography images at the cutting planes shown in *red lines* in B-1 and B-2 reveal a large ventricular septal defect and prominent outlet septum. **c** and **d** Transthoracic echocardiography with color Doppler shows regurgitation of the right-sided atrioventricular valve (mitral valve) (*arrow heads*), with a peak velocity of 5.0 m/second. **e** A three-dimensional model of the cardiac computed tomography at a similar plane to that in (**b**) (as shown in E-1) is shown. The outflows to the aorta and pulmonary trunk are separated by a prominent outlet septum. The anterior mitral leaflet straddles the ventricular septum and is anchored to the ventricular septum (*arrows*). **f** A three-dimensional model of the cardiac computed tomography at a plane similar to that in Figs. 1B-1 and 3B-1 (as shown in F-1) is shown. The mitral leaflet (*arrows*) is anchored close to the pulmonary valve (*arrow heads*). There is a significant malalignment between the ventricular septum and outlet septum. (Supplementary video is available in Additional files 1 and 2). *AO* aorta, *LA* left atrium, *LV* left ventricle, *MV* mitral valve, *OS* outlet septum, *PT* pulmonic trunk, *RA* right atrium, *RV* right ventricle, *TV* tricuspid valve, *VS* ventricular septum

**Fig. 4:**
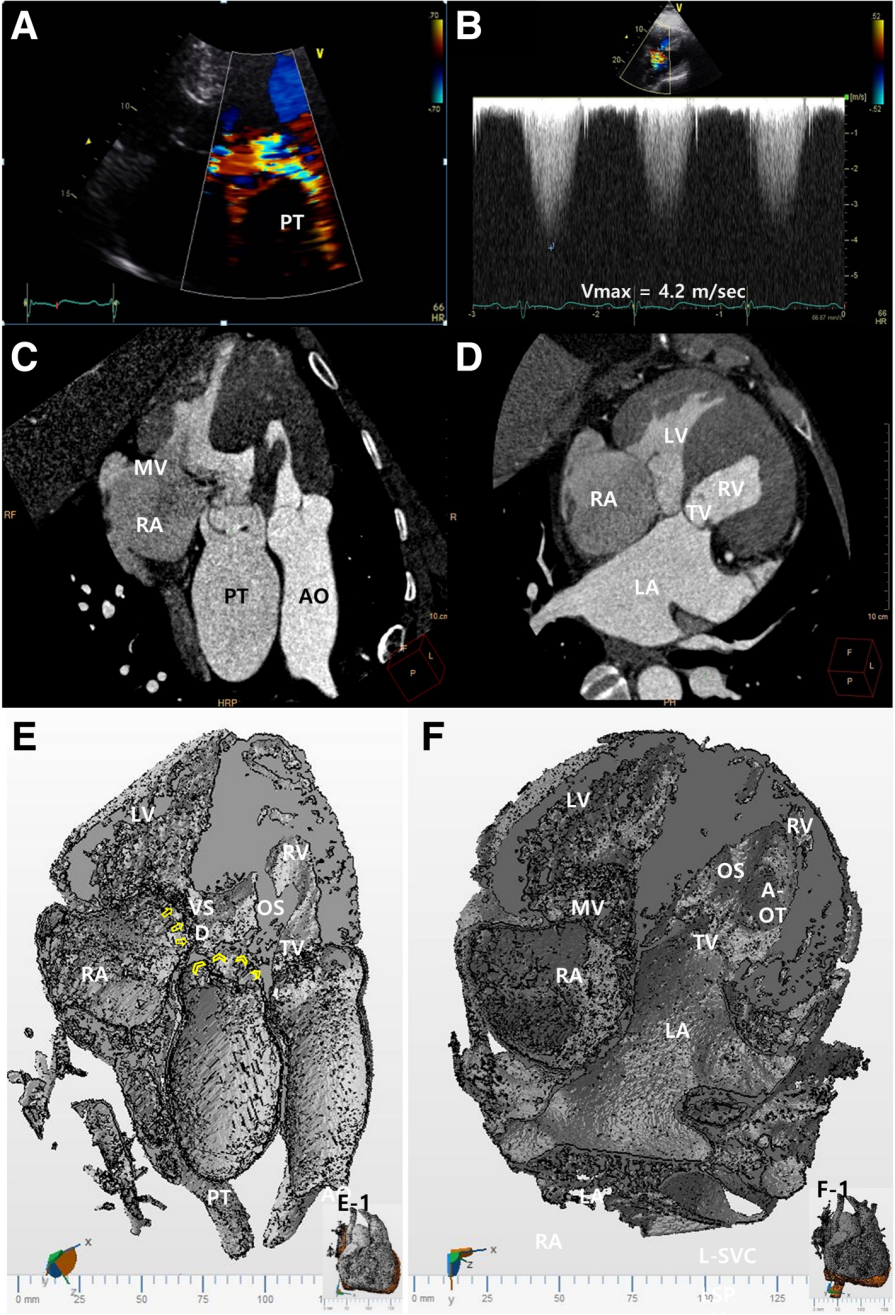
**a** and **b** Transthoracic echocardiography with color Doppler detected a severe pulmonic stenosis, with a peak velocity of 4.2 m/second. **c** Cardiac computed tomography image shows the pulmonic trunk overriding and straddling the ventricular septum. The anterior leaflet of the mitral valve is attached to the ventricular septum near the pulmonary valve. The mitral valve together with the outlet septum of the right ventricle caused the subvalvular pulmonary stenosis. The subaortic part is apparently stenotic in this view. **d** Cardiac computed tomography image also shows the subvalvular structure of the tricuspid valve connected to the ventricular septum. **e** A three-dimensional model of the cardiac computed tomography at a plane of (**c**) (as shown in E-1) shows detailed anatomy of the sub-pulmonary structure and its relation to the ventricular septum. **f** A three-dimensional model of the cardiac computed tomography at a plane similar to that in (**d**) (as shown in F-1) shows the widely open aorta outflow tract. The aortic outflow is separated from the stenotic pulmonary outflow tract by a prominent outlet septum. (Supplementary video is available in Additional files 3 and 4). *AO* aorta, *A-OT* aorta outflow tract, *LA* left atrium, *LV* left ventricle, *MV* mitral valve, *OS* outlet septum, *PT* pulmonic trunk, RA right atrium, *RV* right ventricle, *TV* tricuspid valve, *VSD* ventricular septum defect

A large VSD was also detected by TTE and CCT (Fig. 3a, b). While the left-sided atrioventricular (tricuspid) valve of the systemic ventricle was free of regurgitation, a moderate amount of regurgitant flow, with a peak velocity of 5.0 m/second, was noticed at the right-sided atrioventricular (mitral) valve (Fig. 3c, d), which we assumed was the reason why our patient was diagnosed as having tricuspid regurgitation and severe PAH at the primary hospital. However, we also detected pulmonic stenosis, with a maximal velocity of 4.2 m/second (Fig. 4a, b), even with the normal morphology and opening of the pulmonic valve (Fig. 1a).

Pressure measurement of each chamber by cardiac catheterization demonstrated almost equalization of both the ventricles; the systolic/diastolic/mean pressure of the LV and RV were 114/12/78 mmHg and 120/13/80 mmHg, respectively. Importantly, while the pressure of the aorta was 117/76/94 mmHg, the pulmonary arterial pressure was 36/17/24 mmHg, suggesting the protection of pulmonary vasculature by the presence of severe pulmonic stenosis. Oxygen saturation at the superior and inferior vena cava was 72.3% and 69.2%, respectively, but there was unexpectedly high oxygen saturation (93.9%) at the LV (pulmonic ventricle) near the VSD. Oxygen saturation at the pulmonary artery returned to 88.0% and the saturation at the aorta was 96.9%.

We thought it necessary to assess the detailed morphological features to explain the anatomic substrates of the pulmonary stenosis and the discrepancy in the oxygen saturation data at the right-sided LV. Detailed morphological analysis of the CCT and three-dimensional modeling and printing data were applied to understand the mitral/tricuspid valves and the VSD (Figs. 3e, f and 4e, f; supplementary video is available in Additional file 1). The pulmonary trunk was shifted to the left and overrode the ventricular septum to become a double outlet RV relation of the arterial trunks. However, the outflows to the aorta and pulmonary trunk were separated by a prominent outlet septum (Fig. 3e; supplementary video is available in Additional file 2). Because of the prominent outlet septum, the actual interventricular communication was smaller at the lower end of the outlet septum than at the real VSD (Fig. 3e, f; supplementary video is available in Additional file 3). In addition, there was a straddling of the mitral valve, with a chordal attachment of the mitral valve to the outlet septum of the RV near the pulmonary valve (Fig. 4c). The straddling mitral valve and the outlet septum together formed a severe subvalvular pulmonic stenosis (Fig. 4c, e, f; supplementary video is available in Additional file 4). In addition, the subvalvular structure of the tricuspid valve was connected to the interventricular septum (Fig. 4d). We interpreted these anomalies to reflect the mitral valve straddling over the posteriorly positioned VSD and hypertrophied outlet septum, which together could explain the functional and anatomical sub-pulmonary stenosis and a small amount of shunt flow through the large VSD. Based on these results, we decided to continue medical management, as well-balanced systemic/pulmonary circulations would be maintained with this particular combination of cardiac lesions.

Fifteen days after discharge, our patient visited our emergency room for chest pain, similar to the pain that occurred a month prior. Electrocardiography demonstrated atrial fibrillation with a rapid ventricular response. After direct current cardioversion, his rhythm was converted to sinus rhythm and his chest pain was relieved. He was subsequently started on aspirin and amiodarone and has been asymptomatic for 3 years.

## Discussion and conclusions

We reported an adult case of hemodynamically well-balanced ccTGA with a large VSD, and subvalvular pulmonic stenosis. A detailed anatomical understanding based on TTE, CCT, and three-dimensional printing provided explanation of the functional and anatomical subvalvular pulmonary stenosis and a small amount of shunt flow through the large VSD.

Adult patients with ccTGA present with diverse clinical features, depending on the associated anomalies. In rare examples of ccTGA, there are no other cardiac defects; such individuals may survive over 50 years [3]. Much commoner examples are those with associated defects that must be surgically corrected in childhood to allow survival. Survival of unoperated ccTGA with a VSD and pulmonic stenosis in the fifth decade is exceptional; therefore, it is worthwhile to understand the functional and morphological features of the present patient that allowed him to survive for such a long period. More importantly, it is necessary to have an optimal management plan for this peculiar group of adult congenital heart diseases.

In the present case, the initial clinical question was whether our patient had PAH. Although the presence of a large VSD in adult congenital heart disease is a reasonable suggestion of PAH, a detailed assessment, including cardiac catheterization, denied the presence of PAH and suggested the protection of the pulmonary vasculature by the presence of a severe pulmonic stenosis. Anatomic substrates of a pulmonic stenosis in ccTGA can be valvular or subvalvular. Subvalvular stenosis can be muscular or fibrous, and related to muscular hypertrophy, the presence of a fibrous shelf on the septum, or aneurysmal dilation of fibrous tissue derived from the interventricular component of the membranous septum or atrioventricular valve [4]. It is important to characterize the nature of the stenosis so that any further intervention of the lesion can be planned. Our case had a normal-sized pulmonary valve and good coaptation of the leaflets. In addition, there was a composite subvalvular stenosis of the straddling mitral valve, leftward shift of the pulmonary valve, and hypertrophied/malaligned outlet septum to separate the outflows to the aorta and pulmonary trunk. Mitral valve straddling occurs in hearts with double inlet ventricles or a double outlet RV (including a Taussig–Bing anomaly), as well as in criss-cross hearts. Some rare examples are in cases of ccTGA [5–8]. Pulmonary outflow obstruction in ccTGA not only protects the pulmonary arterial bed, but also protects the left-sided tricuspid valve and RV from failure [9]. In this situation, the morphologic LV has a trained effect as that with pulmonary artery banding; the increased left ventricular pressure compresses the septum, maintaining the tricuspid valve leaflet coaptation. As a result, a reduction in tricuspid regurgitation occurs which leads to improved morphologic RV function and keeps the systemic atrioventricular valve competent [10].

In summary, the presence of a stable sub-pulmonary stenosis with a normal pulmonary valve was the key feature of the present patient. The VSD was functioning as a defect that was smaller than its actual size in this balanced-pressure condition of the ventricles. As this combination is expected to remain constant, we therefore decided that there was no urgent need for intervention in this patient. The follow-up for 3 years supports our expectations; however, heart failure will require cardiac transplantation if other conditions, such as coronary arterial problems, become complicated in the future.

Every patient has a different combination of cardiac defects, and the significance of each individual defect should be analyzed. The observed combination of defects does not always indicate stability. However, we think it important to perform an accurate three-dimensional anatomical evaluation, as well as a functional assessment of the lesions. CCT and three-dimensional assessment of the data were valuable in the present case.

## Additional files

Additional file 1: A three-dimensional model for the overview of the congenitally corrected transposition of the great arteries is shown. (MP4 13393 kb)

Additional file 2: A three-dimensional model of the vertical view of the congenitally corrected transposition of the great arteries is shown. (MP4 11924 kb)

Additional file 3: A three-dimensional model of the detailed anatomy of the sub-pulmonary structure and its relation to the ventricular septum is shown. (MP4 11268 kb)

Additional file 4: A three-dimensional model of the widely open aortic outflow tract is shown. The aortic outflow is separated from the stenotic pulmonary outflow tract. (MP4 5819 kb)

## Data Availability

Not applicable.

## Abbreviations

CCT: Cardiac computed tomography
ccTGA: Congenitally corrected transposition of the great arteries
LV: Left ventricle
PAH: Pulmonary arterial hypertension
RV: Right ventricle
TTE: Transthoracic echocardiography
VSD: Ventricular septal defect
